# Supplementary figures and images for: The evolutionary maintenance of Lévy flight foraging
Source: PLoS Comput Biol. 2022 Jan 18;18(1):e1009490. doi: 10.1371/journal.pcbi.1009490 (PMC8797186; doi:10.1371/journal.pcbi.1009490)

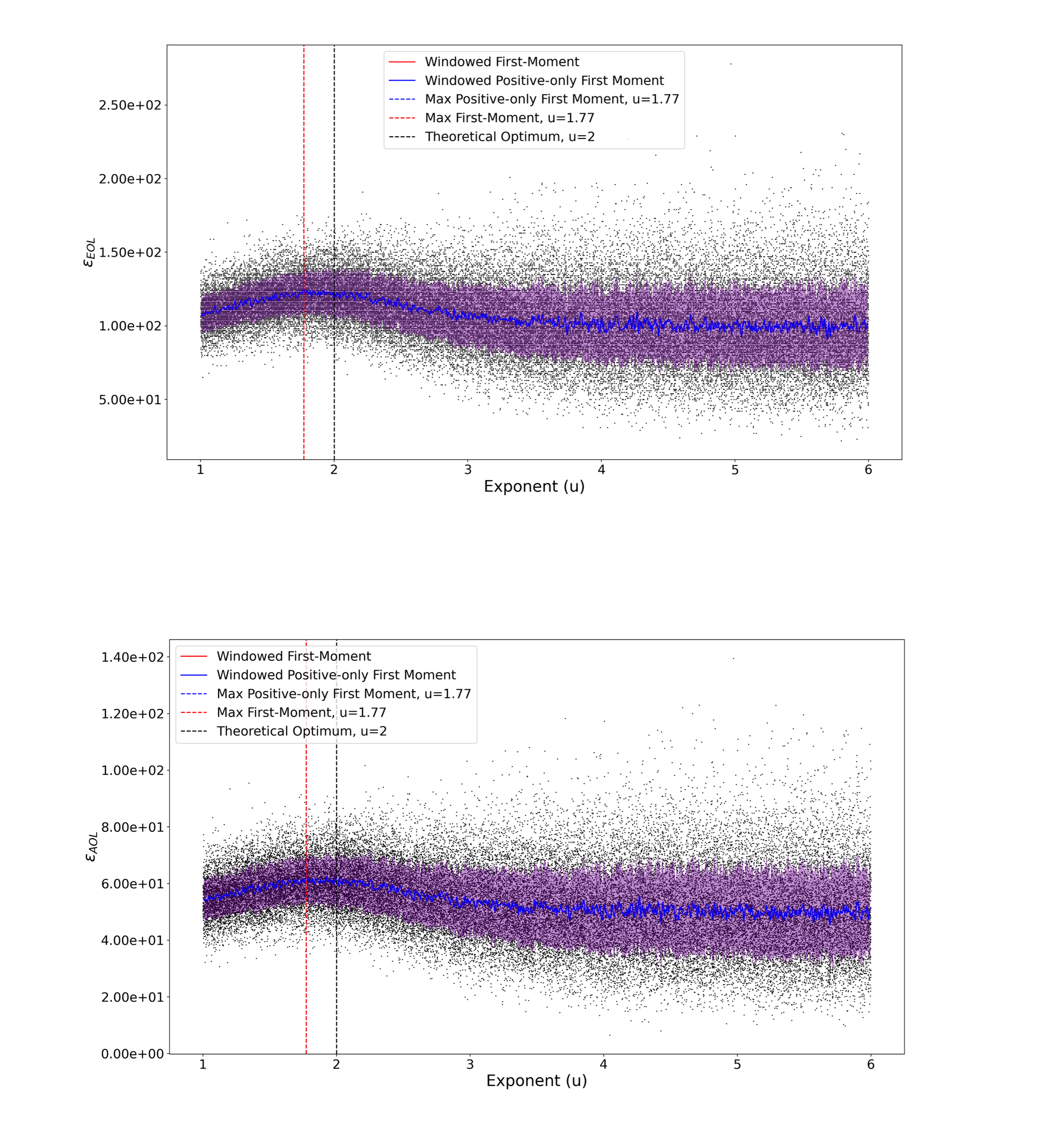

Supplement: S1 Fig — (TIF) [file pcbi.1009490.s001.tif]

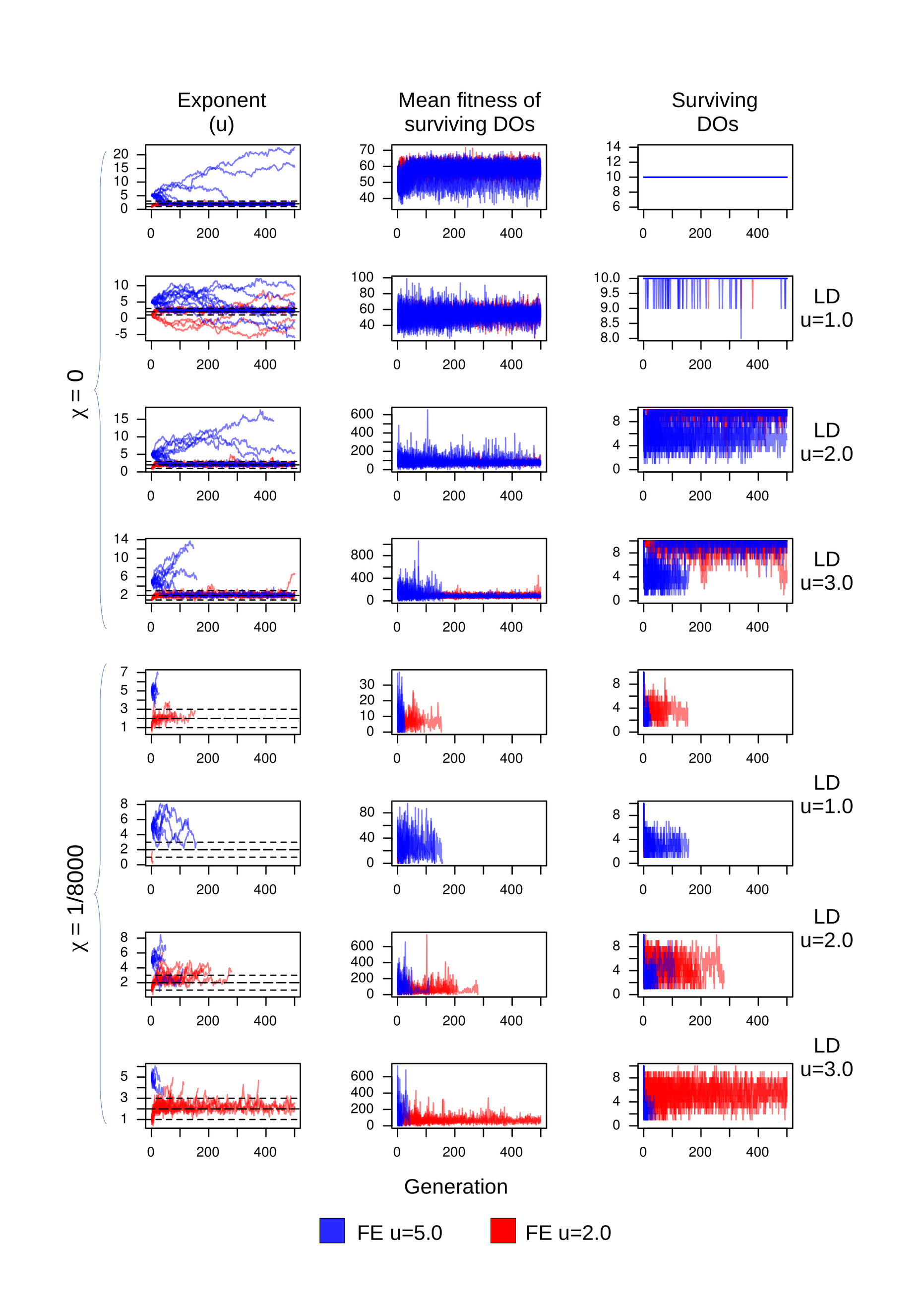

Supplement: S2 Fig — (TIF) [file pcbi.1009490.s002.tif]
