# Supplementary material for: The evolutionary maintenance of Lévy flight foraging
Source: PLoS Comput Biol. 2022 Jan 18;18(1):e1009490. doi: 10.1371/journal.pcbi.1009490 (PMC8797186; doi:10.1371/journal.pcbi.1009490)
Supplement: S1 Appendix — (PDF) [file pcbi.1009490.s003.pdf]

Proposition

Both of a DO's EOL and AOL energy can be computed from s, λ, ξ_λ, $\alpha\xi_\lambda$, d_λ, αd_λ, for any cost χ.

Proof

The energy of a DO at time t is ϵ_t, and for $t = 0$, $\epsilon_0 = 0$. When speed $s = 1$, moving a distance of one consumes exactly one timestep. Thus, if $d_{t+1} = d_t + \delta_t$ and $s = 1$, then $\delta_t = 1$ and $d_t = t \; \forall \; t$. The DO's energy is updated by,

$$\begin{aligned} \epsilon_{t+1} &= \epsilon_t + e_t - \chi\delta_t \\ &= \epsilon_t + e_t - \chi \end{aligned} \tag{1}$$

By letting $\xi_t = \sum_{\tau=0}^{t} e_\tau$ be the sum of resource entries found at time t since birth, the energy at the end of a DO's lifespan, ϵ_{EOL}, can be described as:

$$\epsilon_{EOL} = \epsilon_\lambda = \sum_{t=0}^{\lambda}(e_t - \chi) = \xi_\lambda - \lambda\chi \tag{2}$$

where the value of ϵ_{EOL} with $\chi = 0$ is ξ_λ, and the value of ϵ_{EOL} for a $\chi \neq 0$ model is a simple linear transformation of the zero-cost model. The energy calculation for an AOL model is the sum of Eq 1 over λ:

$$\begin{aligned} \epsilon_{AOL} &= \frac{1}{\lambda}\sum_{t=0}^{\lambda}\epsilon_t \\ &= \frac{1}{\lambda}\sum_{t=0}^{\lambda}\sum_{\tau=0}^{t}(e_\tau - \chi) \\ &= \frac{1}{\lambda}\sum_{t=0}^{\lambda}((\sum_{\tau=0}^{t} e_\tau) - t\chi) \\ &= \frac{1}{\lambda}\sum_{t=0}^{\lambda}\xi_t - \frac{\chi}{\lambda}\sum_{t=0}^{\lambda} t \\ &= \frac{1}{\lambda}\alpha\xi_\lambda - \frac{\chi}{\lambda}\frac{\lambda(\lambda+1)}{2} \end{aligned} \tag{3}$$

Thus, for $\chi = 0$ the ϵ_{AOL} model is the average of ξ_t over a lifespan, and when $\chi \neq 0$, ϵ_{AOL} is a linear transformation of the zero-cost model. Speeds of $s > 1$ result in $\delta_t > 1$

as increasing the speed of a DO reduces the time it takes to traverse the environment. The extreme case is where $s \geq n/2$, in which case a move of any length is traversed in one timestep, and d_λ is the sum of λ move lengths pulled from a DO's S over the DO's lifespan. The generalization of Eqs 2 and 3 for any speed is then achieved by tracking the distance a DO has traveled. Thus, Eq 2 becomes,

$$\epsilon_{EOL} = \epsilon_\lambda = \sum_{t=0}^{\lambda}(e_t - \chi\delta_t) = \xi_\lambda - d_\lambda\chi \tag{4}$$

and Eq 3 becomes,

$$\begin{aligned}
\epsilon_{AOL} &= \frac{1}{\lambda}\sum_{t=0}^{\lambda}\epsilon_t \\
&= \frac{1}{\lambda}\sum_{t=0}^{\lambda}\sum_{\tau=0}^{t}(e_\tau - \chi\delta_\tau) \\
&= \frac{1}{\lambda}\sum_{t=0}^{\lambda}\sum_{\tau=0}^{t}e_\tau - \frac{\chi}{\lambda}\sum_{t=0}^{\lambda}\sum_{\tau=0}^{t}\delta_\tau \\
&= \frac{1}{\lambda}\sum_{t=0}^{\lambda}\xi_t - \frac{\chi}{\lambda}\sum_{t=0}^{\lambda}d_t \\
&= \frac{1}{\lambda}\alpha\xi_\lambda - \frac{\chi}{\lambda}\alpha d_\lambda
\end{aligned} \tag{5}$$

and models with $\chi \neq 0$ remain a simple linear transformation of $\chi = 0$ models, with the additional requirement of recording d_λ and αd_λ for speeds of $s > 1$. ■
