# Supplementary material for: The evolutionary maintenance of Lévy flight foraging
Source: PLoS Comput Biol. 2022 Jan 18;18(1):e1009490. doi: 10.1371/journal.pcbi.1009490 (PMC8797186; doi:10.1371/journal.pcbi.1009490)
Supplement: Attachment — Submitted filename: author_responses.pdf [file pcbi.1009490.s006.pdf]

Response to reviewers comments on: The evolutionary maintenance of Lévy flight foraging

Thank-you very much for the helpful review. I have addressed and implemented all of the proposed changes and suggestions. Changes in the revised manuscript are highlighted in yellow.

Reviewer 1

" There is only one point that I did not understand enough: the use of geometric rather than arithmetic means. We all know that there are different kinds of means, including harmonic means. But after page 5 there is no more mention of geometric means until page 22. If the authors can explain this point better it would be quite useful to the reader." + "As mentioned above was not able fully to understand the difference between geometric and arithmetic mean fitness in this context. Perhaps the authors can further explain this a little bit better."

Response: The arithmetic mean and geometric mean are methods of measuring fitness. This is a fairly well fleshed-out concept within evolutionary biology. Simply put, reproduction is a series of multiplicative events, thus it makes more sense to measure fitness by the geometric mean. A simple example: given the following two repeating lists,

$$A = \{2, 2, 2, 2, 2, 2...\} \quad \& \quad B = \{3, 1, 3, 1, 3, 1...\}$$

by the artihmetic mean, $A == B$. By the geometric mean, $A > B$; thus, A reduces fitness variance. This idea is described very nicely in Simons, 2011; Fig. 1; https://doi.org/10.1098/rspb.2011.0176. I've made changes to line 363 and lines 365-368, as well as citing Simons, 2011 paper to increase the reader's understanding.

" Is the terminology of intrinsic vs. extrinsic hypotheses new? This should be clearly stated. I have only heard of these previously referred to as the adaptationist vs. the emergentist viewpoints, and so forth."

Response: This terminology has been used at least once before, for example, in ref. 30 (Sims, 2019). In the interest of scientific clarity, the introduction now states "(the intrinsic or adaptionist hypothesis)" and "(extrinsic or emergentist hypothesis)" (lines 17 & 19, and lines 438 & 446, respectively).

Reviewer 2

"It is said in the abstract that "Lévy flight is a type of random walk that models the behaviour of many phenomena across a multiplicity of academic disciplines". I disagree with this sentence since LF are a natural phenomenon and not models. I suggest instead 'Lévy flight is a type of random walk present in the behaviour of many natural phenomena across a multiplicity of academic disciplines.'"

Response: Agreed, with the slight tweak "that characterizes the behaviour of many natural phenomena studied across a multiplicity of academic disciplines".

"Even though the possibility of the emergent nature of Lévy flight is mentioned, its treatment is somewhat sloppy. I suggest in order to give more punch to the paper and to show that the authors handle well the literature, that the following papers on deterministic walks be read and discussed either at the introduction or the conclusions:[Ref1] Lima, Gilson F., Alexandre S. Martinez, and Osame Kinouchi. "Deterministic walks in random media." Physical Review Letters 87.1 (2001): 010603.[Ref2] Boyer, Denis, et al. "Scale-free foraging by primates emerges from their interaction with a complex environment." Proceedings of the Royal Society B: Biological Sciences 273.1595 (2006): 1743-1750."

Response: Both papers do not seem to be testing the Lévy flight foraging hypothesis per se (there are no evolutionary components), but I do understand their importance in demonstrating Lévy-like behaviour (or self-avoidance) as being not strictly intrinsic. I discuss this in the following response, too. I've included a brief discussion of these papers in the conclusion (lines 441-447).

"Line 425: "Organisms with Brownian behaviour have a higher probability of re-visiting the same resource and their behaviour may be optimal for shorter lifespans, but with sufficient time they would diffuse into empty space reducing the probability of re-visits. A Lévy-like exponent, however, would have a higher probability of leaving that empty space and eventually encountering resources." There is a paper demonstrating this fact but that has been

ignored:[Ref3] Dannemann, Teodoro, Denis Boyer, and Octavio Miramontes. "Lévy flight movements prevent extinctions and maximize population abundances in fragile LotkaVolterra systems." Proceedings of the National Academy of Sciences 115.15 (2018): 3794-3799."

Response: This paper was very interesting to read and clearly has complementary results. It is now included in the revised manuscript (1. 433-436).

"First sentence of the conclusion is misleading and there is indeed lack of general evidence through the paper to claim this as true: "These results provide evidence that the intrinsic (please correct the typo) hypothesis is a sufficient explanation for Lévy-like behaviour: Lévy flight is the result of selection for behavioural adaptations, rather than an emergent phenomenon due to the encounters within an environments distribution of resources." [Ref2] gives enough theoretical evidence that the distribution of resources modulates the searching strategy resulting in emergent movement patterns. Perhaps the authors would like to explain under which particular and specific circumstances their claim is true and tone it down consequently."

Response: I've encountered [Ref2] before, and I really like that paper. True, it does provide evidence for emergent Lévy behaviour, although it is not strictly a test of the Lévy flight foraging hypothesis (because in [Ref2], "the forager knows the location and size of all targets in the system." whereas in Lévy flight, a forager has no information about the distribution of resources), and the conclusion has been toned down accordingly (linens 438-447). Typo corrected – thanks.

Additional Changes

The following equation:

$$\epsilon_{AOL} = \sum_{i \in n} \epsilon_{EOL_i} = \frac{n+2}{2k} - \frac{1}{2}$$

Should instead be:

$$\epsilon_{AOL} = \frac{\sum_{i \in n} \epsilon_{EOL_i}}{n} = \frac{n+2}{2k} - \frac{1}{2}$$

This was a typo, and does not affect the other equations.
